# Supplementary material for: Modeling Protein Complexes Using Restraints from Crosslinking Mass Spectrometry
Source: Structure. 2018 Jul 3;26(7):1015–1024.e2. doi: 10.1016/j.str.2018.04.016 (PMC6039719; doi:10.1016/j.str.2018.04.016)
Supplement: Document S1. Figures S1–S3 and Tables S2, S4, and S5 [file mmc1.pdf]

Structure, Volume *26*

Supplemental Information

Modeling Protein Complexes Using Restraints from Crosslinking Mass Spectrometry

Joshua Matthew Allen Bullock, Neeladri Sen, Konstantinos Thalassinos, and Maya Topf

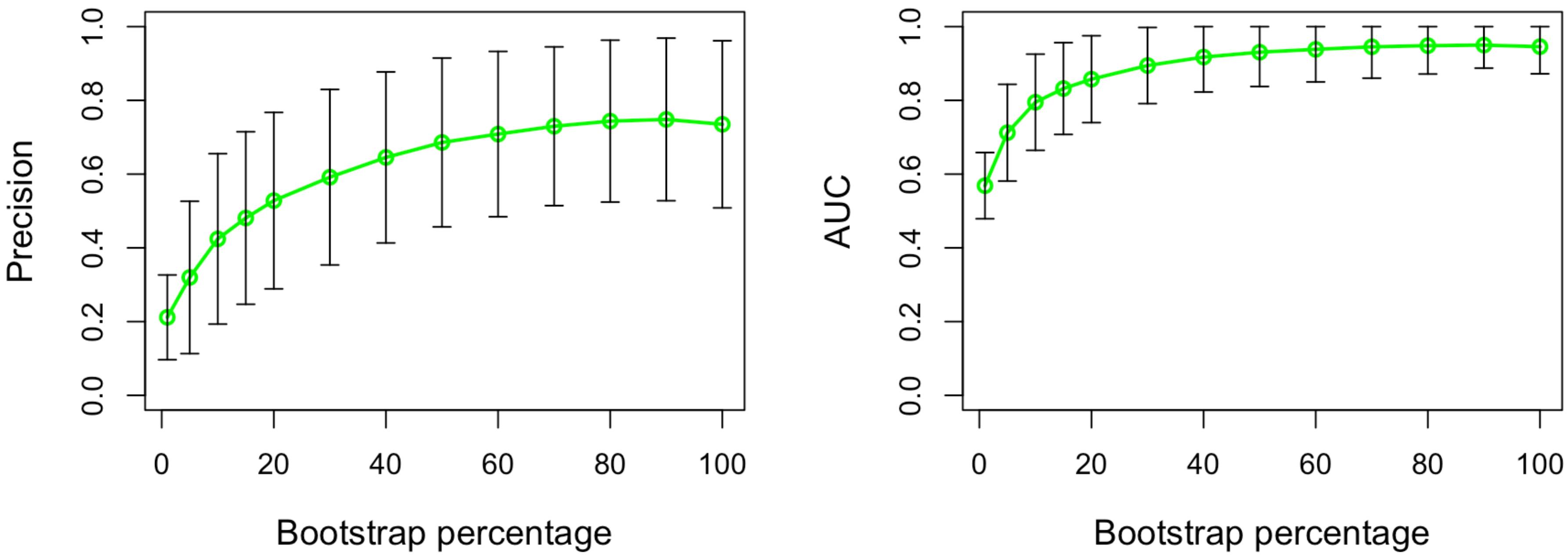

Figure S1 | *Relating to section Performance of cMNXL.* The effect of experimental crosslink recovery on performance. Bootstrapped performance of the benchmark in terms of (A) precision and (B) AUC change as crosslink recovery increases.

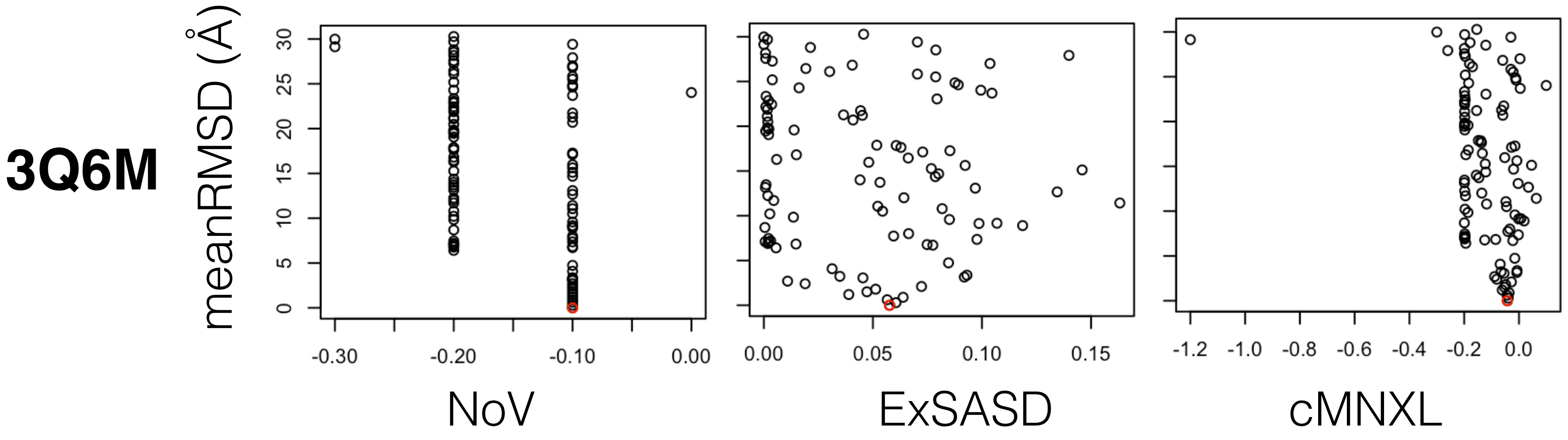

Figure S2 | *Related to section Performance of cMNXL.* Performance plots of 3Q6M showing the general poor performance of each scoring function. The precision of NoV_{inter} is skewed due to many models scoring the same, therefore giving it a high False Positive Rate

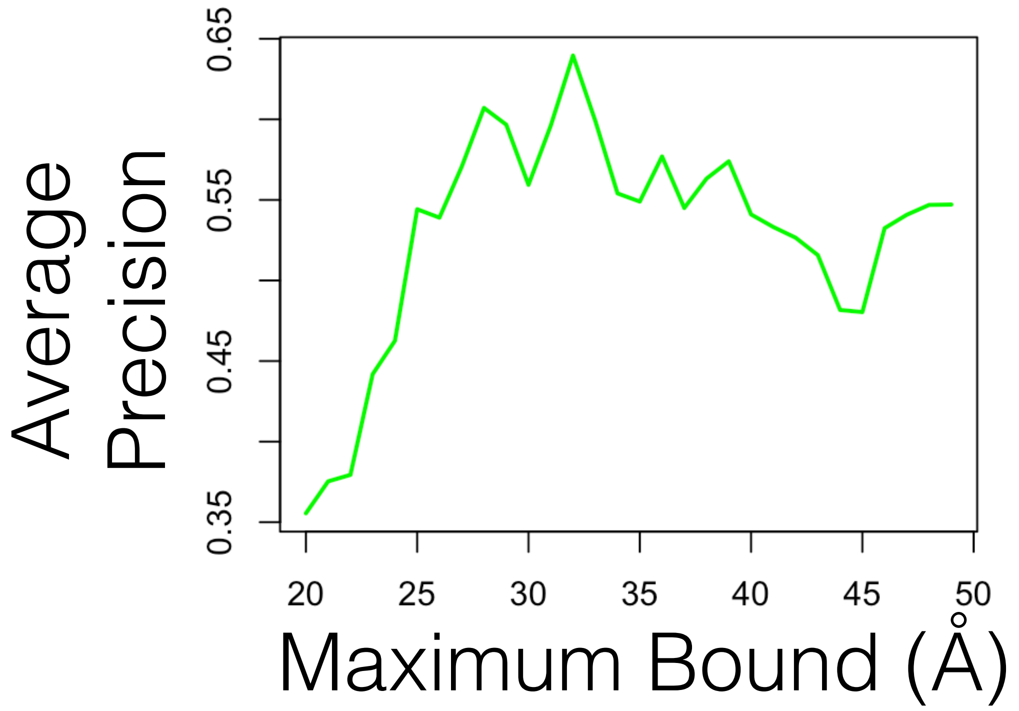

Figure S3 | *Related to section Experimental and Theoretical Cross-links.*
NoV score performance on the experimental benchmark at different maximum bounds. Peak precision is observed at 32Å

PDB	$NoNA_{intra}$			$NoNA_{inter}$			NoV			ExSASD			cMNXL			NoV_{ED}		ExED		$cMNXL_{ED}$	
	Prec	AUC	FPR	Prec	AUC	FPR	Prec	AUC	FPR	Prec	AUC	FPR	Prec	AUC	FPR	Prec	AUC	Prec	AUC	Prec	AUC
**3Q6M**	0.092	0.511	0.898	0.125	0.500	0.864	0.200	0.786	0.427	0.000	0.534	0.114	0.000	0.679	0.114	0.200	0.714	0.000	0.482	0.000	0.617
**3DFQ**	0.206	0.537	0.883	0.326	0.531	0.844	0.677	0.940	0.115	0.800	0.959	0.026	0.800	0.966	0.026	0.761	0.950	1.000	0.999	1.000	1.000
**1I10**	0.201	0.531	0.872	0.322	0.506	0.846	1.000	0.997	0.000	1.000	0.999	0.000	1.000	0.999	0.000	1.000	1.000	1.000	1.000	1.000	1.000
**1UJZ**	0.142	0.534	0.860	0.147	0.534	0.860	0.384	0.843	0.333	0.500	0.927	0.058	0.500	0.927	0.058	0.174	0.669	0.200	0.839	0.300	0.865
**2PSN**	0.100	0.524	0.103	0.231	0.596	0.816	0.635	0.955	0.102	0.800	0.969	0.023	0.500	0.940	0.057	0.477	0.927	0.400	0.915	0.300	0.884
**1IRI**	0.182	0.506	0.877	0.290	0.500	0.852	0.900	0.963	0.012	0.600	0.935	0.049	0.900	0.962	0.012	0.750	0.955	0.400	0.910	0.300	0.918
**1F05**	0.115	0.533	0.875	0.116	0.511	0.875	0.299	0.742	0.157	0.100	0.633	0.102	0.200	0.732	0.091	0.482	0.917	0.000	0.512	0.300	0.891
**1U8F**	0.310	0.525	0.846	0.316	0.531	0.846	1.000	1.000	0.000	1.000	0.999	0.000	1.000	1.000	0.000	1.000	1.000	1.000	0.998	1.000	1.000
**1JEQ**	0.390	0.500	0.900	0.475	0.514	0.817	0.809	0.936	0.098	0.900	0.982	0.000	0.900	0.970	0.000	0.716	0.868	1.000	0.941	1.000	0.950
**Avg.**	0.193	0.522	0.790	0.261	0.525	0.847	0.656	0.907	0.138	0.633	0.882	0.041	0.644	0.908	0.040	0.618	0.889	0.556	0.844	0.578	0.903

Table S2 | *Related to Figure 2*
Precision, AUC and FPR (False Positive Rate) for each benchmark case in the Experimental benchmark when scoring with cMNXL score and each constituent scoring term using both ED and SASD.

PDB	cMNXL		F Score (10 Å)		combined (10Å)		F Score (15Å)		combined (15Å)		F Score (20Å)		combined (20Å)	
	Prec	AUC	Prec	AUC	Prec	AUC	Prec	AUC	Prec	AUC	Prec	AUC	Prec	AUC
**3Q6M**	0.000	0.679	1.000	0.997	1.000	0.997	1.000	0.999	1.000	0.996	1.000	0.998	1.000	0.996
**3DFQ**	0.800	0.966	1.000	0.992	1.000	0.999	1.000	0.996	1.000	0.999	1.000	0.993	1.000	0.999
**1I10**	1.000	0.999	1.000	1.000	1.000	1.000	1.000	1.000	1.000	1.000	1.000	1.000	1.000	1.000
**1UJZ**	0.500	0.927	0.600	0.784	0.600	0.859	0.500	0.702	0.500	0.795	0.500	0.616	0.500	0.726
**2PSN**	0.500	0.940	0.900	0.850	0.900	0.936	0.900	0.859	0.800	0.938	0.800	0.856	0.800	0.939
**1IRI**	0.900	0.962	0.900	0.909	0.900	0.956	0.900	0.908	0.900	0.954	0.900	0.904	0.900	0.956
**1F05**	0.200	0.732	0.600	0.857	0.700	0.875	0.600	0.819	0.500	0.849	0.500	0.781	0.500	0.812
**1U8F**	1.000	1.000	1.000	1.000	1.000	1.000	1.000	1.000	1.000	1.000	1.000	1.000	1.000	1.000
**1JEQ**	1.000	0.975	1.000	0.944	1.000	0.976	1.000	0.943	1.000	0.973	1.000	0.940	1.000	0.972
**Avg.**	0.656	0.909	0.889	0.926	0.900	0.955	0.878	0.914	0.856	0.945	0.856	0.899	0.856	0.933

Table S4 | *Related to Figure 7*
Precision and AUC of cMNXL, F-score at 10Å, 15 Å and 20 Å resolution, and the combined score for each case in the Experimental benchmark.

PDB file	Modifications
3Q6M	Chain C - res 615 inserted to make identical to other chains
3DFQ	None
1I10	None
1UJZ	None
2PSN	None
1IRI	None
1F05	None
1U8F	None
1JEQ	437-454(A), 430-483(B), 269-323(B) removed

Table S5 | *Related to section Protein Complex Benchmarks.* Modifications made to each protein in the benchmark to facilitate model generation.
