# Supplementary material for: Modeling Protein Complexes Using Restraints from Crosslinking Mass Spectrometry
Source: Structure. 2018 Jul 3;26(7):1015–1024.e2. doi: 10.1016/j.str.2018.04.016 (PMC6039719; doi:10.1016/j.str.2018.04.016)
Supplement: Document S2. Article plus Supplemental Information [file mmc5.pdf]

Structure

Resource

Modeling Protein Complexes Using Restraints from Crosslinking Mass Spectrometry

Graphical Abstract

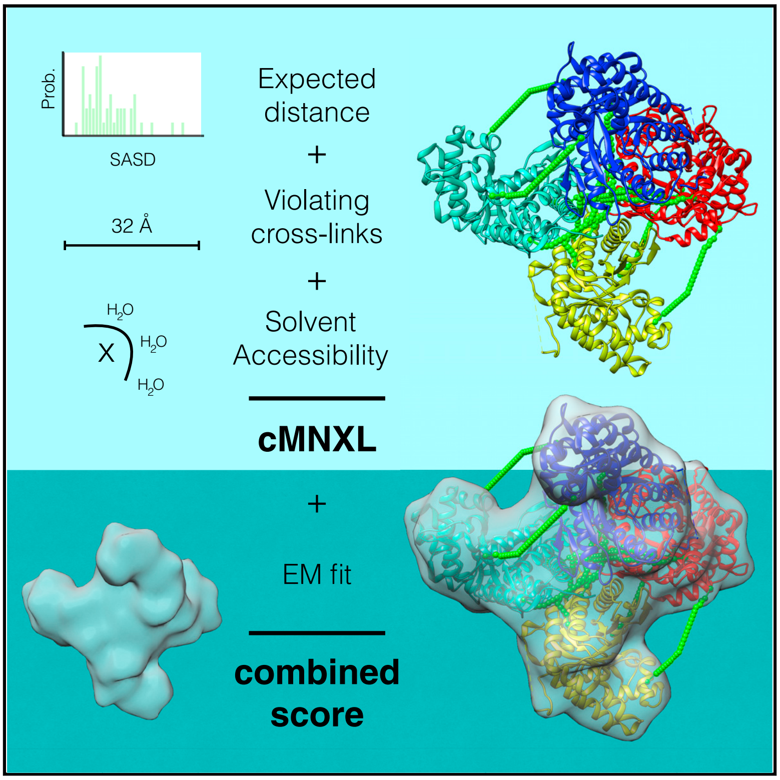

Highlights

- A scoring function that outperforms more commonly used crosslink scoring methods
- A comparison of methods to calculate the distance between crosslinked residues
- Combining low-resolution 3D-EM and crosslinking information improves scoring

Authors

Joshua Matthew Allen Bullock,
Neeladri Sen,
Konstantinos Thalassinos, Maya Topf

Correspondence

m.topf@cryst.bbk.ac.uk

In Brief

Bullock et al. present a scoring function for modeling protein complexes based on crosslink data (cMNXL) that outperforms other functions currently in use. They also combine this method with a 3D-EM fitting score to create a combined scoring function that outperforms each score alone.

Bullock et al., 2018, Structure *26*, 1015–1024
July 3, 2018 © 2018 The Author(s). Published by Elsevier Ltd.
https://doi.org/10.1016/j.str.2018.04.016

CellPress

CellPress

Modeling Protein Complexes Using Restraints from Crosslinking Mass Spectrometry

Joshua Matthew Allen Bullock,[1] Neeladri Sen,[3] Konstantinos Thalassinos,[1,2] and Maya Topf[1,4,*]
[1]Institute of Structural and Molecular Biology, Birkbeck College, University of London, Malet Street, London WC1E 7HX, UK
[2]Institute of Structural and Molecular Biology, Division of Biosciences, University College London, London WC1E 6BT, UK
[3]Indian Institute of Science Education and Research Pune, Pashan, Pune 411 008, India
[4]Lead Contact
*Correspondence: m.topf@cryst.bbk.ac.uk
https://doi.org/10.1016/j.str.2018.04.016

SUMMARY

Modeling macromolecular assemblies with restraints from crosslinking mass spectrometry (XL-MS) tends to focus solely on distance violation. Recently, we identified three different modeling features inherent in crosslink data: (1) expected distance between crosslinked residues; (2) violation of the crosslinker's maximum bound; and (3) solvent accessibility of crosslinked residues. Here, we implement these features in a scoring function. cMNXL, and demonstrate that it outperforms the commonlyused crosslink distance violation. We compare the different methods of calculating the distance between crosslinked residues, which shows no significant change in performance when using Euclidean distance compared with the solvent-accessible surface distance. Finally, we create a combined score that incorporates information from 3D electron microscopy maps as well as crosslinking. This achieves, on average, better results than either information type alone and demonstrates the potential of integrative modeling with XL-MS and low-resolution cryoelectron microscopy.

INTRODUCTION

Protein complexes play a critical role in the cell, either through transient cell signaling complexes or via the specialized functions of macromolecular machines. Determining the structures of protein complexes is therefore essential for a mechanistic understanding of the cell. Unfortunately, protein complexes can be problematic to study with traditional structural techniques, such as X-ray crystallography, nuclear magnetic resonance (NMR), or cryo electron microscopy (EM), due to their size, heterogeneity, or resistance to crystallization (Leitner et al., 2016; Thalassinos et al., 2013). A recently established paradigm, integrative modeling, sidesteps issues arising from individual structural techniques by integrating orthogonal information from a range of experimental sources (Ward et al., 2013). This information is combined into a scoring function that determines how well a given model satisfies the input information.The sources of information can range from traditional structural methods such as X-ray crystallography, NMR, and 3D-EM (Lasker et al., 2012; Russel et al., 2012; Simon et al., 2010) to methods that generate sparse structural information, such as small-angle X-ray scattering (Michie et al., 2016), fluorescence resonance energy transfer (Bonomi et al., 2014), native mass spectrometry (MS) (Politis et al., 2014), ion-mobility MS (Politis et al., 2010), and Crosslinking MS (XL-MS) (Chen et al., 2010; Erzberger et al., 2014; Leitner et al., 2012).

XL-MS is a technique typically used to generate distance restraints, which can be used for protein modeling. The concept of protein crosslinking has been around a long time, although it is not until recently that its popularity has increased, due to technical advances in detector design (Hu et al., 2005), crosslink spectra identification software (Leitner et al., 2014a; Yang et al., 2012), crosslinking reagents (Ihling et al., 2006; Leitner et al., 2014b; Rivera-Santiago et al., 2015), and modeling methodologies (Ferber et al., 2016; Russel et al., 2012). XL-MS restraints can be used exclusively to generate models, or validate models fitted into medium-resolution cryo-EM maps (Wang et al., 2017) or low-resolution subvolume averages from cryo electron tomography (cryo-ET) (Dodonova et al., 2015).

A typical crosslinking experiment consists of first reacting the protein complex with crosslinker, which covalently binds specific amino acids (either lysine, aspartic or glutamic acid, or cysteine) within a given distance (from 0 to 50 Å) depending on the length of the crosslinker. The crosslinked complex is then digested by proteases and the crosslinked peptides are analyzed and identified via MS (Rappsilber, 2011). From this, one can establish which two amino acids are within the crosslinker maximum bound in the native structure, which can be encoded into distance restraints used for modeling.

Another aspect of crosslinking that is commonly overlooked is that crosslinks can only form on residues that are solvent accessible; therefore, crosslinks can act as a proxy for solvent accessibility. We previously made use of this solvent accessibility information by defining a non-accessible crosslink, i.e., a crosslink that has been experimentally observed but where one or both of the crosslinked residues are non-solvent accessible in the model. Incorporating this solvent accessibility information was shown to be very beneficial to the modeling of protein monomers (Bullock et al., 2016).

The distance between two crosslinked residues is usually calculated in one of two ways. The most common method is the Euclidean distance (ED), i.e., a direct line between the two

This is an open access article under the CC BY license (http://creativecommons.org/licenses/by/4.0/).

crosslinked residues. By virtue of its simplicity, it is a very quick calculation to perform; however, as this does not take into account the fact that a crosslinker cannot travel through protein mass, it can be inaccurate. This is in contrast to the solvent-accessible surface distance (SASD), defined as the shortest path between two residues across the surface of the protein (Kahraman et al., 2011), which can be calculated using the software Jwalk (Bullock et al., 2016). Additional accuracy can be obtained by considering the flexibility of crosslinked residues' side chains (Degiacomi et al., 2017).

Previously, when crosslinking restraints have been used for scoring models of protein monomers and complexes the most common approach has been to use only violation of the maximum bound of the crosslinker (number of violations [NoV]). This scoring can be done either via a step function, i.e., a crosslink is either violating or not (Leitner et al., 2012; Politis et al., 2014; Thalassinos et al., 2013), a smoothed scoring function (Belsom et al., 2015; Hofmann et al., 2015) or a probabilistic Bayesian approach (Russel et al., 2012). In all these cases, the only crosslink information used pertains to the crosslinker maximum bound. The expected distance between crosslinked residues (taken from an experimentally observed distribution) can also be used to sample and score models, again implemented in a Bayesian framework in the software XL-MOD (Ferber et al., 2016). It is also possible to use crosslinking data as a proxy for solvent accessibility, which has been previously used in a ROSETTA-based docking protocol (Kahraman et al., 2013).

To improve upon existing crosslink scoring regimes, here we combine the three potential types of modeling restraint encoded in crosslinking information: (1) the maximum bound of crosslinker; (2) the expected distance between crosslinked residues; and (3) the solvent accessibility information of crosslinked residues. Previously, we incorporated these three sources into the scoring function Matched and Non-accessible Crosslink (MNXL) score, and showed it to outperform the NoV method when modeling protein monomers (Bullock et al., 2016). Here we extended the MNXL scoring function to score protein complexes (cMNXL) by incorporating all of the previous information as well as handling intra-subunit and inter-subunit crosslinks differently, in order to maximize modeling performance. We then tested our new scoring function on a simulated benchmark of 68 protein dimers and a separate benchmark of 9 protein complexes, with associated experimental crosslinks taken from XLinkDB2.0 (Schweppe et al., 2016), and compared it with the performance of using either the maximum bound or expected distance alone. We were also able to compare the effects on modeling performance when using SASD or ED to measure the distance between crosslinked residues. Finally, to investigate how crosslinking can be integrated with other structural techniques such as cryo-EM or -ET, we combined cMNXL with 3D-EM density information to generate a combined score, which adds mutual complementary modeling information.

RESULTS

Theory

We updated our previous scoring function to create cMNXL. cMNXL is made up of the number of non-accessible (NoNA) feature of both intra- and inter-subunit crosslinks and the number of violations and expected SASD of inter-subunit crosslinks (NoV and ExSASD, respectively).

The cMNXL score is defined as follows:

$$cMNXL = ExSASD + NoV + 3 \times (NoNA_{inter} + NoNA_{intra}),$$

where *ExSASD* is the expected SASD between inter-subunit crosslinked residues, *NoV* is the number of inter-subunit crosslinks that violate the theoretical maximum bound of the crosslinker, *NoNA* is the number of crosslinks that are non-accessible (i.e., one or both of the crosslinked residues is not solvent accessible), and the subscripts *inter* and *intra* refer to inter-subunit and intra-subunit crosslinks, respectively. During the study, the score from each of these terms was calculated separately and a systematic investigation into the different weighting was performed in order to return the best results (see STAR Methods). The optimum scoring regime for each crosslink feature is described below.

ExSASD: if it is possible to calculate the SASD in the model and the SASD is below the maximum bound, it is scored for inter-subunit crosslinks as follows:

$$ExSASD[SASD] = \begin{cases} N(21.92,\ 4.87) & SASD \leq 32\ \text{Å} \\ 0 & else \end{cases},$$

Where *N* is a normal distribution fitted to the distribution of SASDs for inter-subunit crosslinks under 32 Å taken from the XLdb (Bullock et al., 2016).

In the comparison between SASD and ED, *ExED* is substituted for *ExSASD*. *ExED* is scored as follows:

$$ExED[ED] = \begin{cases} N(18.35,\ 4.11) & ED \leq 30\ \text{Å} \\ 0 & else \end{cases},$$

where N is a normal distribution fitted to the distribution EDs for inter-subunit crosslinks under 30 Å taken from the XLdb (Bullock et al., 2016; Leitner et al., 2012).

NoV: if it is possible to calculate the SASD in the model but the SASD exceeds the crosslinker maximum bound, it is scored as follows:

$$NoV[SASD] = \begin{cases} -0.1 & SASD > 32\ \text{Å} \\ 0 & else \end{cases},$$

or in the case of ED,

$$NoV[ED] = \begin{cases} -0.1 & ED > 30\ \text{Å} \\ 0 & else \end{cases}.$$

NoNA: if no SASD for a pair of either inter- or intra-crosslinked residues can be calculated, because one or both of the crosslinked residues are no longer solvent accessible, this crosslink is defined as non-accessible, respectively, and is scored a flat penalty of -0.1. cMNXL score belongs to the set $\{cMNXL \in \mathbb{R}\}$.

cMNXL Scoring Workflow

To score models of protein complexes using crosslink data it is necessary to generate a test dataset, i.e., the calculation of SASDs between all solvent exposed lysine residues. The test

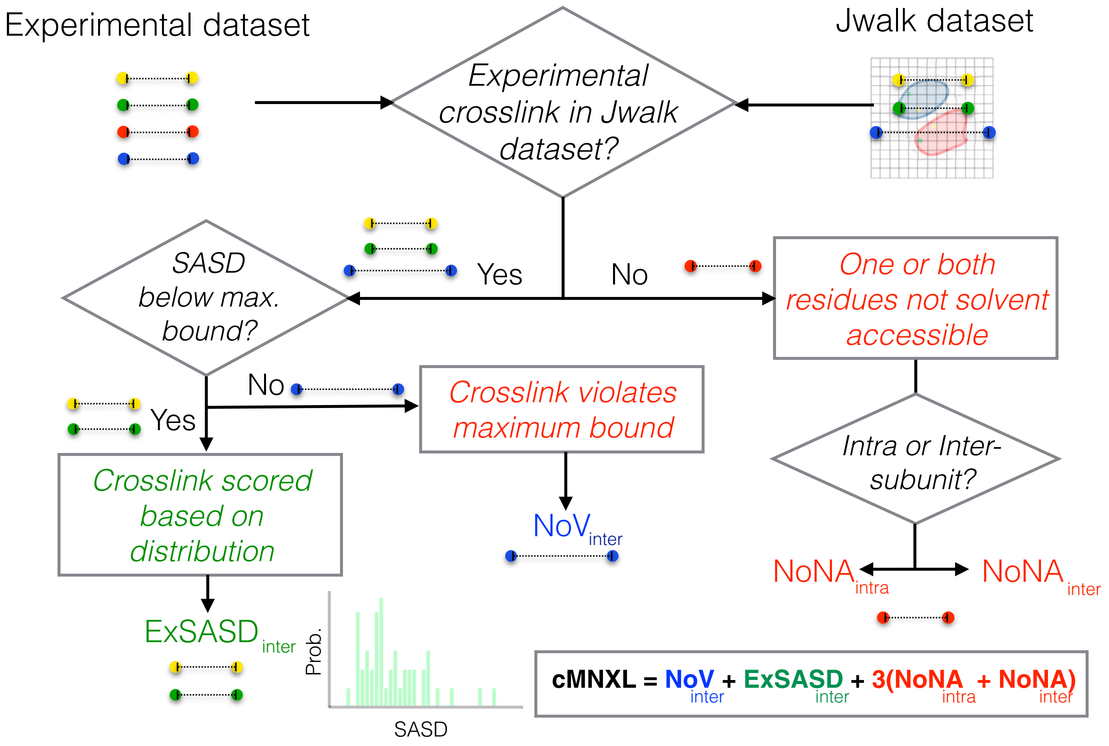

Figure 1. Flowchart Detailing the Scoring Workflow of cMNXL

cMNXL is the total of the scores for each individual experimental crosslink.

dataset is then compared against either the experimental MS dataset (crosslinks taken from the XLinkDB2.0 [Schweppe et al., 2016]) or a theoretical MS dataset (i.e., all the SASDs calculated with Jwalk [Bullock et al., 2016] under the maximum bound of 32 Å). Each crosslinked pair of residues, in either the experimental or theoretical MS datasets, is then compared against the test dataset based on which the crosslinks are scored. The score for each crosslink is then totaled into a final score for each model (Figure 1).

Performance of cMNXL

Theoretical Benchmark

To statistically evaluate this scoring function, we tested cMNXL on a theoretical benchmark of 68 protein dimers, each with 100 models at a range of qualities. The models were scored using theoretical crosslinks. To replicate a more realistic scenario, we bootstrapped crosslinks at 15% recovery with a minimum of 2 inter-subunit crosslinks (see STAR Methods).

Overall, cMNXL achieves an average precision of 0.560 and average area under the curve (AUC) of 0.901 (Figure 2 and Table S1). This is a significant improvement over the more commonly used NoV score, which has an average precision of 0.438 and AUC of 0.875 (p values 4.11e−12 and 2.835e−06, respectively). cMNXL also has a significantly lower false-positive rate (FPR) than NoV (0.050 versus 0.207) making cMNXL a more reliable score ($p < 2.2e{-16}$).

There is a range of performance across the benchmark. Cases that can be modeled successfully tend to have high frequency of lysine residues on the surface, for example PDB: 1QA9 or 1FQJ, which contain 27 and 38 lysines across the complex, respectively (Figure 3 and Table S1). Cases that perform badly include PDB: 1FFW, in which one subunit has only two lysines and therefore one subunit position cannot be triangulated against the other (Figure 3). If the complex is too small for the length of restraint used, the results will also be bad, as in the case of PDB: 2OOB, because significant deviations from the native structure can be made without violating any of the distance restraints (Figure 3). Testing cMNXL at different levels of recovery confirms that an increase in crosslink recovery improves performance, although this performance starts to plateau at a theoretical recovery of ~50% (Figure S1).

The best-performing term of cMNXL is the *ExSASD* score, which alone achieves an average precision and AUC of 0.529 and 0.897, respectively. This is followed by the NoV term, which achieves a lower precision and AUC of 0.428 and 0.875, respectively. The non-accessible crosslink terms ($NoNA_{intra}$ and $NoNA_{inter}$) perform the least, with low precisions when used on their own (0.128 and 0.161 for $NoNA_{intra}$ and $NoNA_{inter}$, respectively) (Figure 2 and Table S1). However, when combined with the other scoring terms there is a small but significant improvement in precision (from 0.557 to 0.560, $p = 0.0329$) but not in AUC.

Experimental Benchmark

To discover how cMNXL performed with experimental crosslinking data, we then tested cMNXL on a second benchmark of 9 protein complexes, each with 100 models at a range of quality, using experimental crosslinks taken from the XLinkDB2.0.

cMNXL achieves a higher average precision of 0.644 and AUC of 0.908. Although overall on this small benchmark the

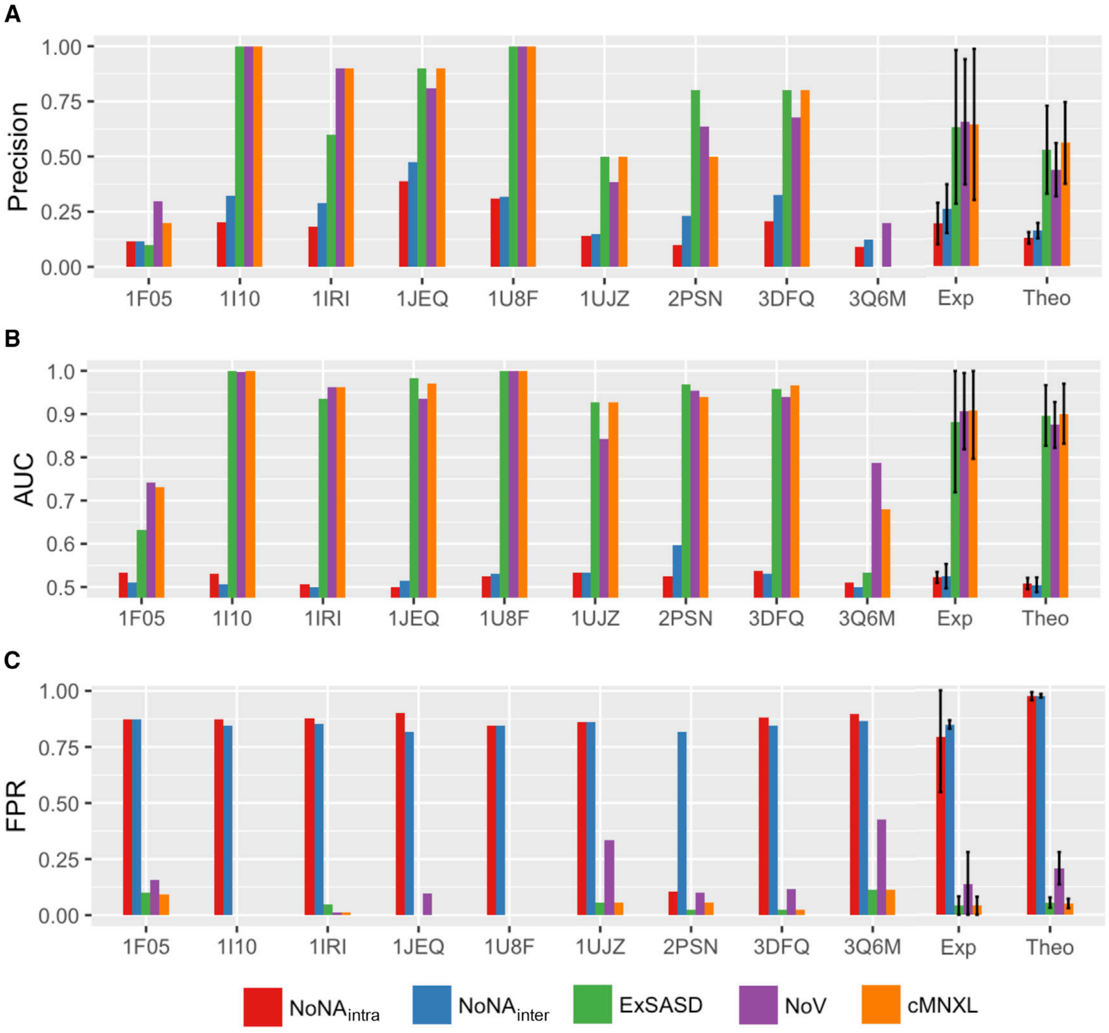

Figure 2. Performance of cMNXL Terms

Bar plots showing the performance of each individual member of the experimental benchmark as well as the average performance of both the experimental (Exp) and theoretical (Theo) benchmarks, when scored with each constituent scoring term of cMNXL, in terms of (A) precision, (B) AUC, and (C) false-positive rate (FPR). The error bars represent the standard deviation.

NoV score performs similarly to cMNXL, with non-significant increase in precision and a non-significant decrease in AUC (0.656 and 0.907 for precision and AUC, respectively), the FPR is significantly worse (from 0.138 to 0.040, p = 0.018). In the 3 out of 9 individual cases where NoV outperforms cMNXL, NoV has a much higher FPR (Figure 2 and Table S2).

Generally across the benchmark, the higher the number of crosslinks, the more successful the modeling, as is the case for PDB: 1U8F, which has 162 crosslinks (42 inter- and 120 intra-subunit) (Figure 4). However, PDB: 1JEQ also performs very well, which is surprising given the lower count of crosslinks (4 inter- and 5 intra-subunit crosslinks). Here, the inter-subunit crosslinks are located in three discrete regions across the protein interface, which triangulates the protein orientation and optimizes the scoring.

When there are not enough inter-subunit crosslinks to triangulate the protein orientation, modeling performance (with either cNMXL or NoV alone) is severely affected. With PDB: 1F05 (Figure 2) there are only 2 inter- and 10 intra-subunit crosslinks. Without a third inter-subunit crosslink to triangulate the orientation, many bad models do not violate any crosslinks at all. The top scoring model is mostly correct in terms of orientation; however, the subunits are rotated so that the two inter-subunit crosslinks are closer together than in the native structure, as this maximizes the ExSASD scoring aspect. PDB: 3Q6M is the worst performing in the benchmark because there are only three inter-crosslinks between three subunits and as a result, large deviations in orientation are tolerated (Figure 4). In this case, the NoV appears to outperform cMNXL; however, this is an artifact of the way precision and AUC are calculated in NoV (see STAR Methods), given that 49 models across a range of mean root-mean-square deviations (RMSDs) (0.00–29.41 Å) only violate one crosslink and as a result all are ranked second. The poor FPR of NoV (0.427) reflects this (Figures 2 and S2).

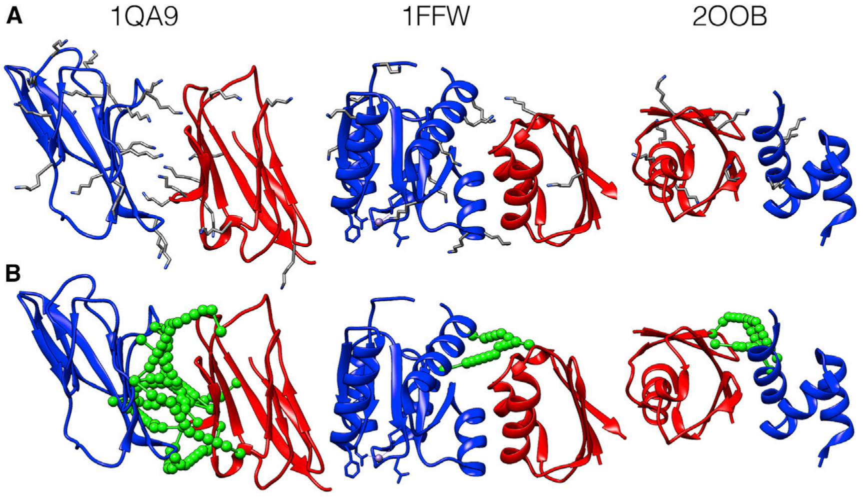

Figure 3. Specific Cases from the Theoretical Benchmark, PDB: 1QA9, 1FFW, and 2OOB

(A) Protein structures with all lysine side-chain atoms are shown in gray.

(B) Protein structures with a representative theoretical crosslink benchmark (sampled at 15% of all possible crosslink combinations under 32 Å) are shown in green.

Comparison of SASD and ED

Next, we compared the effect on modeling performance when using either SASD or ED to calculate the distance between crosslinked residues. While SASD is theoretically more correct, using Jwalk to calculate SASD is around 5 orders of magnitude slower than ED (despite significant speed improvements due to parallelization). This can become prohibitive when dealing with large numbers of models and crosslinks.

We substituted ED for SASD in cMNXL ($cMNXL_{ED}$) to make a direct comparison between the distance types and retain the solvent accessibility information that would otherwise be lost when solely using ED. Upon use of ED, there is no significant change in precision (from 0.560 to 0.542 for cMNXL to $cMNXL_{ED}$, respectively) or AUC (from 0.901 to 0.906 for cMNXL to $cMNXL_{ED}$, respectively) (Figure 5 and Table S1). Looking specifically at ExSASD and substituting in the ED (ExED) also gave only a small non-significant decrease in both precision and AUC. Surprisingly, substituting ED into NoV (NoV_{ED}) and comparing with NoV does result in a significant decrease in performance, in both precision and AUC (p values 1.141e−09 and 2.321e−12 for precision and AUC, respectively).

This trend is generally also observed in the experimental benchmark, where there are non-significant decreases in precision and AUC when switching from cMNXL to $cMNXL_{ED}$ (Figure 5 and Table S2). The performance difference between NoV and NoV_{ED} is larger but also remains insignificant. Surprisingly, the comparison between ExSASD and ExED shows a much larger drop in performance, where the decrease in AUC is significant (0.882–0.844, p = 0.0281). This can be attributed to two benchmark cases, PDB: 1UJZ and 2PSN, whose performances decrease more dramatically (Figure 6). The poor performance of 1UJZ is a result of the protein complex being very small (9.9 and 14.5 kDa each), and therefore the crosslink restraints tolerate a large range of non-native models. When the ED is used the tolerance is increased further, therefore accepting an even greater range of non-native models (Figure 6A). In the case of 2PSN, the precision drops from 0.800 to 0.400 because 14/18 inter-subunit crosslinks lie very close to the dimer interface, all stemming from either K53 and K59 (Figure 6B). The ExSASD performs well because a large number of the inter-subunit crosslinks have native SASDs very close to the mean SASD value (μ = 21.92 Å). However, this performance halves when using the ExED because the ED distribution does not accurately reflect the distances between crosslinked lysines.

Combination with 3D-EM Data

To explore the power of combining crosslinking and 3D-EM information, we combined cMNXL with the fitness score (F score) from γ-TEMPy (Pandurangan et al., 2015), a genetic algorithm for generating models of protein complexes using 3D-EM density. To this end, we simulated 3D-EM density maps at 10-, 15-, and 20-Å resolution from both the theoretical and experimental benchmarks (see STAR Methods, note that for the experimental benchmark only the crosslinks are "experimental," not the density maps).

Scoring the theoretical benchmark with each scoring function (F score and cMNXL) separately reveals that on average the F score performs significantly better than cMNXL at each resolution in terms of both AUC (p values 4.288e−4, 1.628e−3, and 9.451e−3 for 10, 15, and 20 Å, respectively) and precision (p values 2.2e−16, 3.197e−15, and 4.706e−15 for 10, 15, and 20 Å, respectively). However, the combination of F score with cMNXL significantly improves the average performance at every resolution compared with F score alone in terms of AUC (p values 2.942e−10, 2.976e−11, and 9.747e−13 for 10, 15, and 20 Å, respectively) and precision (p values 1.22e−05, 5.663e−05, and 5.458e−06 for 10, 15, and 20 Å, respectively) (Figure 7 and Table S3).

Crosslinks are especially useful in cases such as PDB: 1XD3 from the theoretical benchmark, where globular subunits are difficult to orientate accurately (Figure 8A). Here, the F score ranks many models with completely incorrect interfaces (*fnat* of 0) in the top 10, resulting in a precision of 0.40. The combination of crosslinking and 3D-EM, even when the crosslinking alone is unable to successfully model the protein complex, improves the orientation of the subunits, increasing the precision to 0.90.

In the experimental benchmark this trend is repeated, with the combined score significantly outperforming the F score at every resolution in terms of average AUC (p values 0.0140, 0.0160, and 0.0161 for 10, 15, and 20 Å, respectively). In terms of precision, the F score alone already performs excellently so there is little room for additional improvement (Figure 7 and Table S4). In benchmark case PDB: 1F05, cMNXL alone performs badly

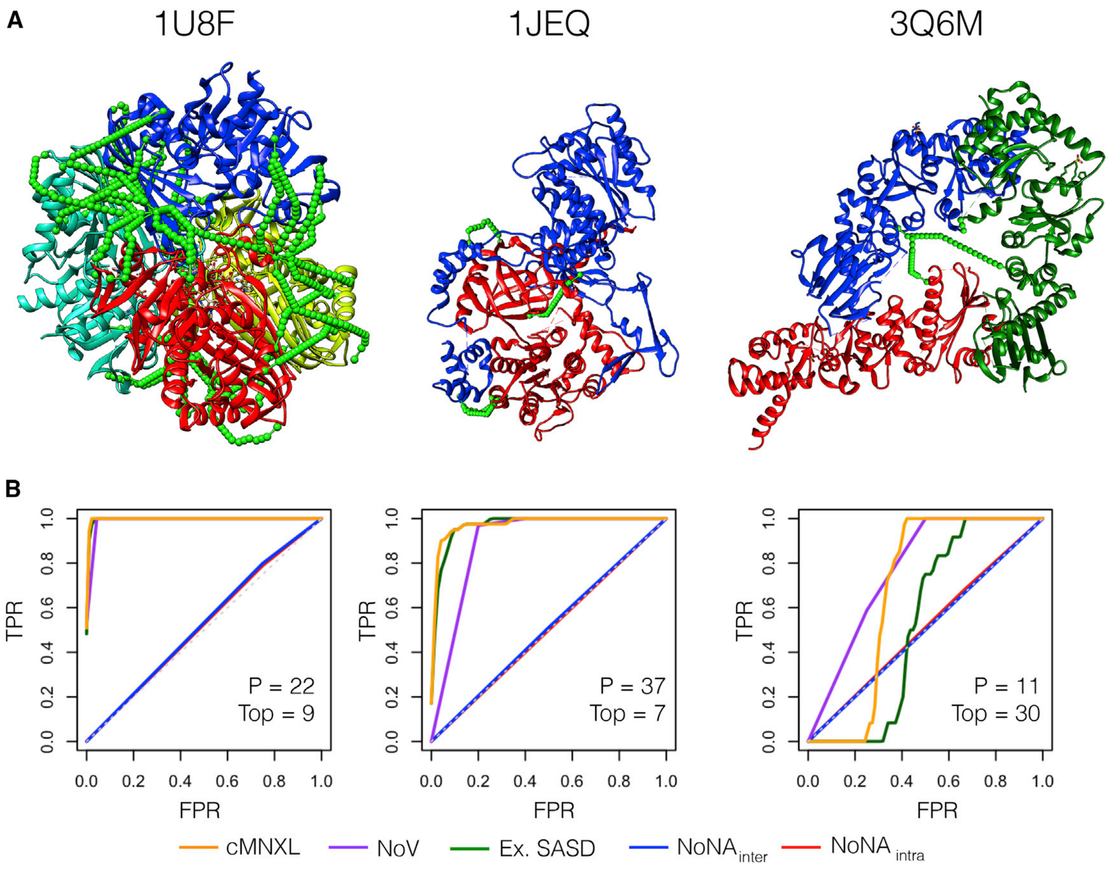

Figure 4. Performance of cMNXL and Its Components Demonstrated on Three Benchmark Cases, PDB: 1U8F, 1JEQ, and 3Q6M

(A) Respective crystal structures of with experimental crosslink SASDs mapped on the surface using Jwalk. Green SASDs pertain to inter-subunit crosslinks.

(B) Respective receiver-operating characteristic (ROC) plots showing the different components of cMNXL: expected SASD (ExSASD), violations (NoV), intra-subunit non-accessible crosslinks ($NoNA_{intra}$), and inter-subunit non-accessible crosslinks ($NoNA_{inter}$). P denotes the number of positive models in the benchmark, and Top denotes the cMNXL rank of the best model based on meanRMSD.

because it has only 2 inter-subunit crosslinks (see above) (Figure 8B). Again, the combined score is able to improve the performance of F score despite the crosslinking alone being insufficient, as one of the models ranked in the top10 by the F score violates a crosslink restraint, which results in a precision increase from 0.600 to 0.700 (F score to combined score, respectively).

DISCUSSION

General Approach to Crosslinking Data

Our previous investigation into modeling monomeric proteins (Bullock et al., 2016) and our current investigation into protein complexes highlights three separate, but complementary, scoring properties of crosslinks: (1) the expected SASD between two crosslinked residues (*ExSASD*); (2) the number of violations of the crosslinker maximum bound between two crosslinked residues (*NoV*); (3) the solvent accessibility of the crosslinked residues (*NoNA*). By making use of all three of these aspects, we were able to create a scoring function that improves upon the commonly used inter-subunit NoV or the expected distance between inter-subunit ExSASD.

We tested our score on both an experimental and a theoretical benchmark. The theoretical benchmark was scored at a 15% recovery rate in order to more accurately replicate an experimental scenario (the average experimental recovery rate of the experimental benchmark was 13.7%). Scanning of the recovery rates shows us, however, that at ~50% recovery the modeling precision begins to plateau. Future technical improvements in the collection of crosslinks should therefore increase the performance of XL-MS modeling.

Lysine crosslinkers are also able to crosslink serine and threonine residues, albeit at much lower frequencies (Sinz et al., 2015). As of yet, no serine or threonine crosslinks are listed in the XLinkDB2.0. However, cMNXL is applicable to any crosslinked residue that is crosslinked using a crosslinker of 11.4 Å length.

Previously we showed that non-accessible crosslinks play an important role in modeling the structure of monomeric proteins (Bullock et al., 2016). In a benchmark containing only protein monomers, *NoNA* contributed the most performance to the scoring function, whereas *NoV* and *ExSASD* contributed only smaller gains. Interestingly, this has not been the case with our current results on protein complexes, where out of all the scoring aspects, *NoNA* contributes the least (and the inter-subunit ones less than the intra-subunit), while both *NoV* and *ExSASD* deliver similar high performance.

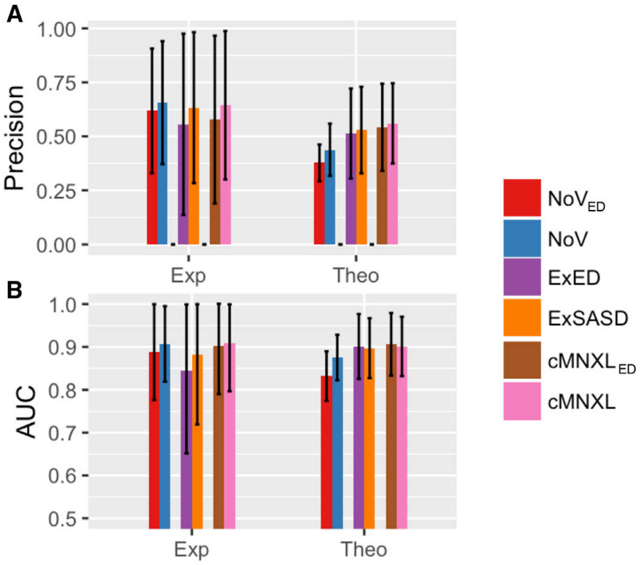

Figure 5. Performance of Scores Using ED versus SASD

Bar plots showing the performance of both the experimental (Exp) and theoretical (Theo) benchmarks, when scored with either NoV, ExSASD, or cMNXL using either ED or SASD, in terms of (A) precision and (B) AUC. The error bars represent the standard deviation.

These results highlight the differences between the requirements of the data for the two types of modeling: proteins versus protein complexes. In modeling complexes using proteins as rigid bodies, distance information from crosslinks can effectively constrain the conformational space as opposed to solvent accessibility (if two rigid bodies are far enough removed, their surfaces would be fully exposed and all solvent accessibility restraints would be satisfied). However, solvent accessibility can in theory guide subunit orientation once rigid bodies reach close proximity (both inter- and intra-), which may explain the small but significant increase in performance seen when incorporating non-accessible crosslinks into cMNXL. This is in contrast to modeling different conformations of protein monomers, whereby the distance between crosslinked residues is unlikely to differ dramatically between models, and backbone flexibility opens up the possibility of burying or exposing different residues. In this instance, non-accessible crosslinks markedly increase the performance while *NoV* and *ExSASD* contribute less (Bullock et al., 2016).

Estimation of the Distance between Crosslinked Residues: SASD versus ED

In our previous study on monomers, the effect of using SASD over ED was pronounced, especially in the comparison between MNXL and NoV$_{ED}$, where the latter's performance collapsed completely (precision of 0.06). However, in this benchmark on protein complexes, the difference in performance is much less marked. This is likely because in protein monomers, physically the crosslink must curve around the protein surface, which creates a larger discrepancy between the ED and SASD. In the case of complexes, there is a higher chance of the crosslink to be more "linear" when connecting two residues, thereby making the ED a reasonable approximation for inter-subunit crosslinker paths in protein complexes. This would be even more so when using crosslinkers shorter than BS3 (e.g., BS2) or zero-length crosslinkers (e.g., EDC). These results again highlight how rigid-body modeling of protein complexes has information requirements different from those of the flexible modeling of protein monomers.

In our theoretical benchmark, the performance between SASD and ED only differs significantly when using the NoV term (i.e., NoV$_{SASD}$ and NoV$_{ED}$). This is similar to the observation made by Kahraman et al. (2013) whereby they found the numbers of models satisfying a threshold of crosslinking restraints to decrease dramatically when switching from NoV$_{ED}$ to NoV$_{SASD}$. This observation contrasts with what is seen in our experimental benchmark, where the performance is significantly different only in the expected distance term (ExED performing worse than ExSASD). However, as shwon, this result is due to two specific cases (PDB: 1UJZ and 2PSN) that perform badly, and is therefore not indicative of a more general trend.

Another consideration when deciding which distance calculation to use is the computational expense of calculating SASD, which is too high to implement in a sampling-based methodology. Possible alternatives include calculating the ED between the Nζ atoms of lysines after considering their flexibility (Degiacomi et al., 2017).

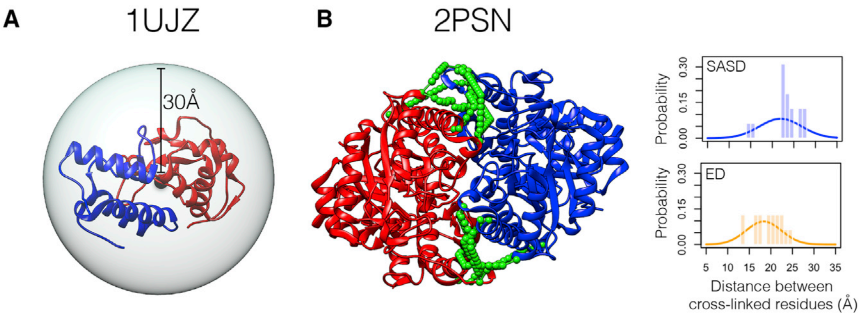

Figure 6. Comparison of Performance between SASD and ED

(A) Crystal structure of benchmark case PDB: 1UJZ placed inside a 30-Å radius sphere centered on the center of mass, demonstrating how ED loses its ability to score small protein complexes.

(B) Left: crystal structure of benchmark case PDB: 2PSN with experimental inter-subunit crosslink SASDs mapped on the surface in green. Inter-subunit crosslinks are clustered in two regions close to the dimer interface. Right: distributions of expected SASD and ED in blue and orange, respectively. Histograms of SASD (blue) and ED (orange) of inter-subunit crosslinks for benchmark case 2PSN overlaid. More SASDs score at the maximum of the expected SASD distribution than EDs score on the expected ED distribution, which results in better modeling performance for ExSASD.

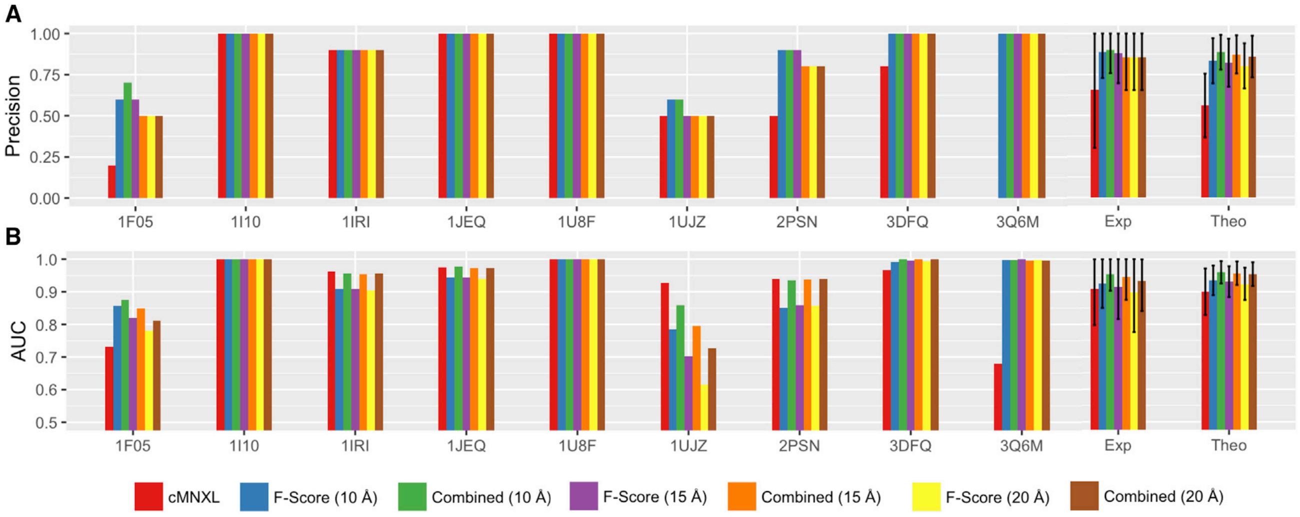

Figure 7. Performance of cMNXL, F Score, and the Combined Score

Bar plots showing the performance of each individual member of the experimental benchmark as well as the average performance of both the experimental (Exp) and theoretical (Theo) benchmarks, when scored with cMNXL, F score, and the combined score using 3D-EM maps at 10, 15, and 20 Å resolution, in terms of (A) precision and (B) AUC. The error bars represent the standard deviation.

Considering the above, if the NoV is the scoring method being used, we recommend using SASD (Kahraman et al., 2013). This is because NoV_{ED} remains the worst crosslink scoring procedure, performing significantly worse than $cMNXL_{SASD}$ and also NoV_{SASD}. If using other types of scoring functions (e.g., cMNXL or expected distance [Ferber et al., 2016]) are used, as the non-significant gain generated from modeling with SASD is offset by its computational expense, we recommend using ED (especially when considering using sampling methods with crosslink information [Erzberger et al., 2014; Ferber et al., 2016]).

Conflicting Crosslinks and False Positives

As the crosslinking experiment happens in solution at room temperature, it is possible that crosslinking datasets are capturing more than one conformation of the complex. This could be a result of the protein complex adopting multiple native

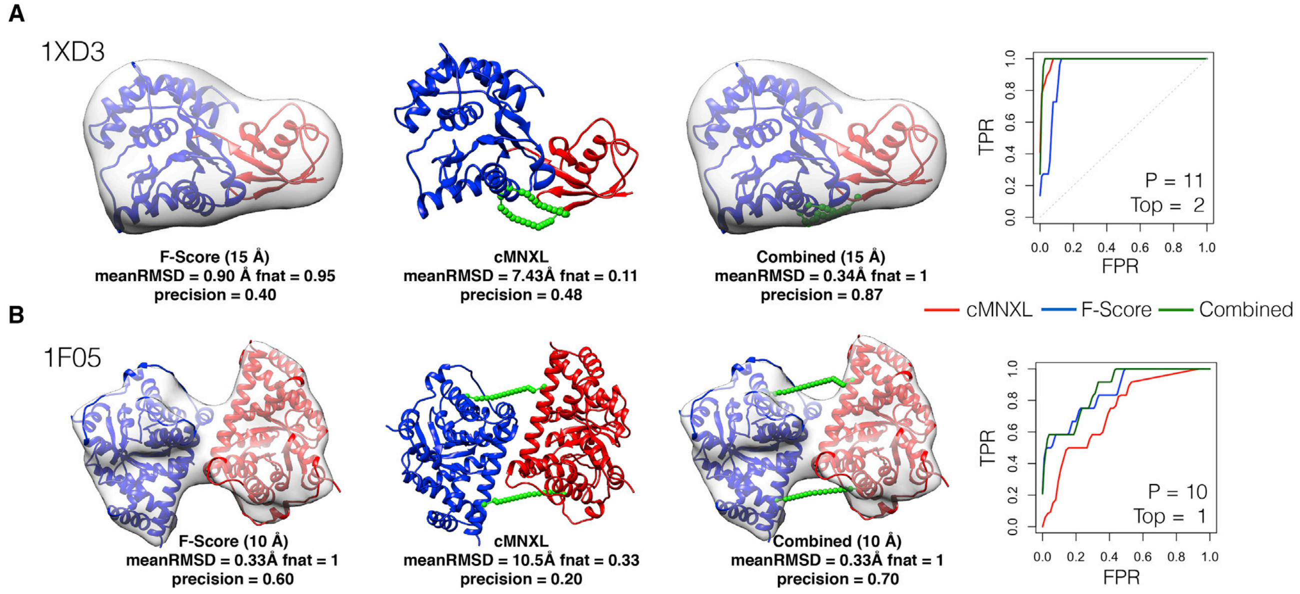

Figure 8. Performance of cMNXL, F Score, and Combined Score

The top-ranked model from the F score, cMNXL, and the combined score, along with corresponding ROC plots of (A) theoretical benchmark case PDB: 1XD3 and (B) experimental benchmark case PDB: 1F05. The native structure and top-ranked F score and combined score models are shown with the native 3D-EM map simulated at 15- and 10-Å resolution for 1XD3 and 1F05, respectively. The top-ranked model from cMNXL and the combined score show the inter-subunit SASDs in green (representative dataset bootstrapped at 15% for 1XD3). The precision shown is the bootstrapped average. Inside the ROC plots, P denotes the number of positive models in the benchmark, and Top denotes the cMNXL rank of the best model based on mean RMSD.

conformations, but it could also be due to crosslinks capturing a partly dissociated complex. Consequently, this might lead to conflicting sets of crosslinks that correspond to (in terms of distance restraints and solvent accessibility information) two or more structural states. Modeling procedures that use crosslinking in the sampling stage are more likely to identify these events, either by specifically processing these events during sampling (Ferber et al., 2016) or by clustering the output to see whether multiple scoring minima are observed (Erzberger et al., 2014). In this study, we used cMNXL only to validate models and, as such, we were unable to deal with conflicting crosslink datasets. Nevertheless, the use of crosslinks to validate models generated by 3D-EM fitting is commonly seen in the literature (Dodonova et al., 2015; Wang et al., 2017).

Additionally, during the crosslink identification process it is likely that a small percentage of the identified crosslinks will be false positives. Experimental methods for assessing the quality of crosslinks are becoming more rigorous (Iacobucci and Sinz, 2017); however, currently the most effective way to incorporate this information is to use the crosslink identification score (e.g., Xquest ID score [Rinner et al., 2008]). Unfortunately, these data are not available in the XLinkDB2.0 or XLDB, so we were unable to include this in cMNXL. However, we plan to explore this in future studies.

Combining Crosslinking and 3D-EM Information

Finally, we wanted to demonstrate that crosslinking and 3D-EM information at resolution range of 10–20 Å are complementary to each other in the modeling process. By combining cMNXL with the fitness score (F score) from γ-TEMPy, we created a combined score that performed better overall on the benchmark than each score alone. Even though the 3D-EM maps were *simulated*, which gave the F score an advantage over the crosslinking data (which was either experimental or was bootstrapped at 15% for the theoretical benchmark, i.e., more realistic than the noise-free EM data), the results confirmed that the combination of crosslinking and 3D-EM information is complementary at all resolutions and can capture subunit orientation better than 3D-EM alone. This is most notable in certain cases such as PDB: 1XD3, where models ranked in the top 10 by the F score have completely incorrect interfaces. Even in cases where the crosslinking information is insufficient to successfully model protein complexes on its own (experimental benchmark case PDB: 1F05), the combination of crosslinking with 3D-EM information still generates an improvement.

Using a combination of crosslinks and 3D-EM data could be especially relevant to applications in cryo-ET where the maps resulting from subtomogram averaging are still often in the intermediate- to low-resolution range (Thalassinos et al., 2013). Combining both *in-cell* crosslinking and cryo-ET should improve the structural characterization of macromolecular complexes within the native environment.

STAR★METHODS

Detailed methods are provided in the online version of this paper and include the following:

- KEY RESOURCES TABLE
- CONTACT FOR REAGENT AND RESOURCE SHARING
- METHOD DETAILS
  - cMNXL Score
  - cMNXL Weighting
  - Protein Complex Benchmarks
  - Experimental and Theoretical Cross-Links
  - 3D-EM Data
- QUANTIFICATION AND STATISTICAL ANALYSIS
  - Measuring Model Accuracy
  - Score Assessment
- DATA AND SOFTWARE AVAILABILITY

SUPPLEMENTAL INFORMATION

Supplemental Information includes three figures and six tables and can be found with this article online at https://doi.org/10.1016/j.str.2018.04.016.

ACKNOWLEDGMENTS

We thank Dr. David Houldershaw for computer support, Dr. Madhusudhan Mallur Srivatsan, Dr. Agnel Joseph, the Topf group, and Thalassinos group for helpful discussions. This work was supported by BBSRC London Interdisciplinary Doctoral Programme (to J.B.), CSIR-SPMF and Newton Bhbabha PhD Placement Fellowship (to N.S.), MRC MR/M019292/1 (to M.T.), and Welcome Trust Collaborative Award (209250/Z/17/Z) (to M.T. and K.T.).

AUTHOR CONTRIBUTIONS

Conceptualization, J.M.A.B., M.T., and K.T.; Methodology, J.M.A.B., N.S., and M.T.; Software, Validation, and Formal Analysis, J.M.A.B.; Investigation, J.M.A.B., N.S., and M.T.; Resources, M.T.; Writing – Original Draft, J.M.A.B.; Writing – Review & Editing, J.M.A.B., M.T., and K.T.; Visualization, J.M.A.B.; Funding Acquisition, M.T. and K.T.

DECLARATION OF INTERESTS

The authors declare no competing interests.

Received: June 8, 2017
Revised: March 5, 2018
Accepted: April 25, 2018
Published: May 24, 2018

REFERENCES

Belsom, A., Schneider, M., Fischer, L., Brock, O., and Rappsilber, J. (2015). Serum albumin domain structures in human blood serum by mass spectrometry and computational biology. Mol. Cell. Proteomics *15*, 1105–1116.

Bonomi, M., Pellarin, R., Kim, S.J., Russel, D., Sundin, B.A., Riffle, M., Jaschob, D., Ramsden, R., Davis, T.N., Muller, E.G.D., et al. (2014). Determining protein complex structures based on a Bayesian model of in vivo Förster resonance energy transfer (FRET) data. Mol. Cell. Proteomics *13*, 2812–2823.

Bullock, J.M.A., Schwab, J., Thalassinos, K., and Topf, M. (2016). The importance of non-accessible crosslinks and solvent accessible surface distance in modelling proteins with restraints from crosslinking mass spectrometry. Mol. Cell. Proteomics *15*, 2491–2500.

Chavez, J.D., Schweppe, D.K., Eng, J.K., and Bruce, J.E. (2016). In vivo conformational dynamics of Hsp90 and its interactors. Cell Chem. Biol. *23*, 716–726.

Chen, Z.A., Jawhari, A., Fischer, L., Buchen, C., Tahir, S., Kamenski, T., Rasmussen, M., Lariviere, L., Bukowski-Wills, J.-C., Nilges, M., et al. (2010). Architecture of the RNA polymerase II-TFIIF complex revealed by cross-linking and mass spectrometry. EMBO J. *29*, 717–726.

Degiacomi, M.T., Schmidt, C., Baldwin, A.J., and Benesch, J.L.P. (2017). Accommodating protein dynamics in the modeling of chemical crosslinks. Structure *25*, 1751–1757.e5.

Dodonova, S.O., Von Appen, A., Hagen, W.J.H., Beck, R., Beck, M., Wieland, F., and Briggs, J.A.G. (2015). A structure of the COPI coat and the role of coat proteins in membrane vesicle assembly. Science *349*, 195–198.

Erzberger, J.P., Stengel, F., Pellarin, R., Zhang, S., Schaefer, T., Aylett, C.H.S., Cimermančič, P., Boehringer, D., Sali, A., Aebersold, R., et al. (2014). Molecular architecture of the 40S•eIF1•eIF3 translation initiation complex. Cell *158*, 1123–1135.

Farabella, I., Vasishtan, D., Joseph, A.P., Pandurangan, A.P., Sahota, H., and Topf, M. (2015). TEMPy: a python library for assessment of 3D electron microscopy density fits. J. Appl. Crystallogr. *48*, 1314–1323.

Ferber, M., Kosinski, J., Ori, A., Rashid, U.J., Moreno-Morcillo, M., Simon, B., Bouvier, G., Batista, P.R., Muller, C.W., Beck, M., et al. (2016). Automated structure modeling of large protein assemblies using crosslinks as distance restraints. Nat. Methods *13*, 515–520.

Herzog, F., Kahraman, A., Boehringer, D., Mak, R., Bracher, A., Walzthoeni, T., Leitner, A., Beck, M., Hartl, F.-U., Ban, N., et al. (2012). Structural probing of a protein phosphatase 2A network by chemical cross-linking and mass spectrometry. Science *9114*, 1348–1352.

Hofmann, T., Fischer, A.W., Meiler, J., and Kalkhof, S. (2015). Protein structure prediction guided by crosslinking restraints—a systematic evaluation of the impact of the crosslinking spacer length. Methods *89*, 79–90.

Hu, Q., Noll, R.J., Li, H., Makarov, A., Hardman, M., and Cooks, R.G. (2005). The Orbitrap: a new mass spectrometer. J. Mass Spectrom. *40*, 430–443.

Iacobucci, C., and Sinz, A. (2017). To be or not to be? Five guidelines to avoid misassignments in cross-linking/mass spectrometry. Anal. Chem. *89*, 7832–7835.

Ihling, C., Schmidt, A., Kalkhof, S., Schulz, D.M., Stingl, C., Mechtler, K., Haack, M., Beck-Sickinger, A.G., Cooper, D.M.F., and Sinz, A. (2006). Isotope-labeled cross-linkers and Fourier transform ion cyclotron resonance mass spectrometry for structural analysis of a protein/peptide complex. J. Am. Soc. Mass Spectrom. *17*, 1100–1113.

Kahraman, A., Malmström, L., and Aebersold, R. (2011). Xwalk: computing and visualizing distances in cross-linking experiments. Bioinformatics *27*, 2163–2164.

Kahraman, A., Herzog, F., Leitner, A., Rosenberger, G., Aebersold, R., and Malmström, L. (2013). Cross-link guided molecular modeling with ROSETTA. PLoS One *8*, e73411.

Lasker, K., Förster, F., Bohn, S., Walzthoeni, T., Villa, E., Unverdorben, P., Beck, F., Aebersold, R., Sali, A., and Baumeister, W. (2012). Molecular architecture of the 26S proteasome holocomplex determined by an integrative approach. Proc. Natl. Acad. Sci. USA *109*, 1380–1387.

Leitner, A., Joachimiak, L.A., Bracher, A., Mönkemeyer, L., Walzthoeni, T., Chen, B., Pechmann, S., Holmes, S., Cong, Y., Ma, B., et al. (2012). The molecular architecture of the eukaryotic chaperonin TRiC/CCT. Structure *20*, 814–825.

Leitner, A., Walzthoeni, T., and Aebersold, R. (2014a). Lysine-specific chemical cross-linking of protein complexes and identification of cross-linking sites using LC-MS/MS and the xQuest/xProphet software pipeline. Nat. Protoc. *9*, 120–137.

Leitner, A., Joachimiak, L.A., Unverdorben, P., Walzthoeni, T., Frydman, J., Förster, F., and Aebersold, R. (2014b). Chemical cross-linking/mass spectrometry targeting acidic residues in proteins and protein complexes. Proc. Natl. Acad. Sci. USA *111*, 9455–9460.

Leitner, A., Faini, M., Stengel, F., and Aebersold, R. (2016). Crosslinking and mass spectrometry: an integrated technology to understand the structure and function of molecular machines. Trends Biochem. Sci. *41*, 20–32.

Lensink, M.F., and Wodak, S.J. (2010). Docking and scoring protein interactions: CAPRI 2009. Proteins *78*, 3073–3084.

Lensink, M.F., and Wodak, S.J. (2013). Docking, scoring, and affinity prediction in CAPRI. Proteins *81*, 2082–2095.

Michie, K.A., Kwan, A.H., Tung, C.S., Guss, J.M., and Trewhella, J. (2016). A highly conserved yet flexible linker is part of a polymorphic protein-binding domain in myosin-binding protein C. Structure *24*, 2000–2007.

Pandurangan, A.P., Vasishtan, D., Alber, F., and Topf, M. (2015). γ-TEMPy: simultaneous fitting of components in 3D-EM maps of their assembly using a genetic algorithm. Structure *23*, 2365–2376.

Pettersen, E., Goddard, T., Huang, C., Couch, G., Greenblatt, D., Meng, E., and Ferrin, T. (2004). UCSF Chimera—a visualization system for exploratory research and analysis. J. Comput. Chem. *25*, 1605–1612.

Politis, A., Park, A.Y., Hyung, S.-J., Barsky, D., Ruotolo, B.T., and Robinson, C.V. (2010). Integrating ion mobility mass spectrometry with molecular modelling to determine the architecture of multiprotein complexes. PLoS One *5*, e12080.

Politis, A., Stengel, F., Hall, Z., Hernández, H., Leitner, A., Walzthoeni, T., Robinson, C.V., and Aebersold, R. (2014). A mass spectrometry-based hybrid method for structural modeling of protein complexes. Nat. Methods *11*, 403–406.

Rappsilber, J. (2011). The beginning of a beautiful friendship: cross-linking/mass spectrometry and modelling of proteins and multi-protein complexes. J. Struct. Biol. *173*, 530–540.

Rinner, O., Seebacher, J., and Walzthoeni, T. (2008). Identification of cross-linked peptides from large sequence databases. Nat. Methods *5*, 315–318.

Rivera-Santiago, R.F., Sriswasdi, S., Harper, S.L., and Speicher, D.W. (2015). Probing structures of large protein complexes using zero-length cross-linking. Methods *89*, 99–111.

Russel, D., Lasker, K., Webb, B., Velázquez-Muriel, J., Tjioe, E., Schneidman-Duhovny, D., Peterson, B., and Sali, A. (2012). Putting the pieces together: integrative modeling platform software for structure determination of macromolecular assemblies. PLoS Biol. *10*, e1001244.

Šali, A., and Blundell, T. (1993). Comparative protein modelling by satisfaction of spatial restraints. J. Mol. Biol. *234*, 779–815.

Schweppe, D.K., Zheng, C., Chavez, J.D., Navare, A.T., Wu, X., Eng, J.K., and Bruce, J.E. (2016). XLinkDB 2.0: integrated, large-scale structural analysis of protein crosslinking data. Bioinformatics *32*, 2716–2718.

Simon, B., Madl, T., Mackereth, C.D., Nilges, M., and Sattler, M. (2010). An efficient protocol for NMR-spectroscopy-based structure determination of protein complexes in solution. Angew. Chem. Int. Ed. *49*, 1967–1970.

Sinz, A., Arlt, C., Chorev, D., and Sharon, M. (2015). Chemical cross-linking and native mass spectrometry: a fruitful combination for structural biology. Protein Sci. *24*, 1193–1209.

Thalassinos, K., Pandurangan, A.P., Xu, M., Alber, F., and Topf, M. (2013). Conformational States of macromolecular assemblies explored by integrative structure calculation. Structure *21*, 1500–1508.

Vasishtan, D., and Topf, M. (2011). Scoring functions for cryoEM density fitting. J. Struct. Biol. *174*, 333–343.

Vreven, T., Moal, I.H., Vangone, A., Pierce, B.G., Kastritis, P.L., Torchala, M., Chaleil, R., Jiménez-García, B., Bates, P.A., Fernandez-Recio, J., et al. (2015). Updates to the integrated protein-protein interaction benchmarks: docking benchmark version 5 and affinity benchmark version 2. J. Mol. Biol. *427*, 3031–3041.

Wang, X., Cimermancic, P., Yu, C., Schweitzer, A., Chopra, N., Engel, J.L., Greenberg, C., Huszagh, A.S., Beck, F., Sakata, E., et al. (2017). Molecular details underlying dynamic structures and regulation of the human 26S proteasome. Mol. Cell. Proteomics *16*, 840–854.

Ward, A.B., Sali, A., and Wilson, I.A. (2013). Integrative structural biology. Science *339*, 913–915.

Yang, B., Wu, Y.-J., Zhu, M., Fan, S.-B., Lin, J., Zhang, K., Li, S., Chi, H., Li, Y.-X., Chen, H.-F., et al. (2012). Identification of cross-linked peptides from complex samples. Nat. Methods *9*, 904–906.

STAR★METHODS

KEY RESOURCES TABLE

REAGENT or RESOURCE	SOURCE	IDENTIFIER
Deposited Data		
Experimental cross-linking data	this paper	Table S6
Theoretical benchmark	cMNXL Theoretical benchmark	http://topf-group.ismb.lon.ac.uk/Software.html
Experimental benchmark	cMNXL Experimental benchmark	http://topf-group.ismb.lon.ac.uk/Software.html
Software and Algorithms		
cMNXL	cMNXL version 1.0	http://topf-group.ismb.lon.ac.uk/Software.html

CONTACT FOR REAGENT AND RESOURCE SHARING

Further information and requests for data should be directed to and will be fulfilled by the Lead Contact, Maya Topf (m.topf@cryst.bbk.ac.uk)

METHOD DETAILS

cMNXL Score

The theory behind the cMNXL score is described in the main text. cMNXL is freely available as a python package from http://topf-group.ismb.lon.ac.uk/Software.html and in the TEMPy software (http://tempy.lon.ismb.ac.uk)

cMNXL Weighting

The weighting of each scoring aspect in the final cMNXL scoring function was reached via a systematic scan of weights for each aspect from 0 to 1 (in steps of 0.1), simultaneously. This scan was performed on the larger theoretical benchmark (see below) and the weights that gave the highest precision were chosen.

Protein Complex Benchmarks

There are two benchmarks in this study. One theoretical, which consists of 68 protein dimers, taken from rigid body dataset created by Vreven *et al*. (Vreven et al., 2015) and a second experimental, consisting of 9 complexes (2-mers to 4-mers) containing proteins only (i.e. not including complexes consisting of DNA/RNA) taken from the XLinkdb2.0 (Schweppe et al., 2016).

The dimer dataset was created in order to have a dataset large enough to generate statistically significant conclusions. The cross-links for the dimers were generated theoretically, taking a 15% recovery, i.e. 15% of all possible theoretical cross-links (see below). The minimum criteria for selecting a set of theoretical cross-links was for it to have at least 2 inter-subunit cross-links. The choice of 15% recovery reflects the typical recovery from an XL-MS experiment (the average recovery of the experimental benchmark is 13.7%). This process was repeated 1000 times and the average precision and AUC taken. The performance of cMNXL on this benchmark was also tested at 1/5/10/20/30/40/50/60/70/80/90 and 100% recovery (Figure S1).

The experimental benchmark, taken specifically from XLinkDB2.0 based on the in-cell cross-linking datasets (Herzog et al., 2012) and (Chavez et al., 2016), was used to test the scoring functions on genuine experimental cross-linking data. These datasets were chosen as they are the only datasets that have corresponding PDB structures, solved using X-ray crystallography. The minimum criteria for selecting a protein complex from the databases was for it to have at least 2 inter-subunit cross-links and at least 5 cross-links in total. The number of subunits in each complex range from 2 to 4.

Using TEMPy (Farabella et al., 2015), for each protein complex, subunits were iteratively translated and rotated a random distance and angle between -5 and 5 Å and 0 to 180°, respectively. Models were then filtered for clashes, allowing a maximum of 20 main-chain atom clashes. 100 models were selected to be as close to uniform distribution of meanRMSD (see Measuring Model Accuracy) as possible. In order to generate near-native models for some of the complexes, loop regions had to be cleaved (Table S5).

For the purposes of this study, all homo-complexes in the benchmark have been treated like hetero-complexes, i.e. all identical subunits and associated cross-links were treated as unique subunits, with individual cross-links specified to each subunit (this is currently not possible experimentally). In order to determine if a homo-complex cross-link should be intra- or inter-subunit, the SASDs of all the possible combinations were calculated and the lowest SASD was chosen (if no SASD under 32 Å was present in the native the lowest SASD was still taken). All reciprocal crosslink combinations were reproduced.

Experimental and Theoretical Cross-Links

All the cross-links in the experimental datasets (Table S6) were either BS3 or DSS – both with a linker arm of 11.4 Å. SASDs and Euclidean distances (EDs) between cross-linked residues were calculated using Jwalk (Bullock et al., 2016). Theoretical cross-links are defined as all the lysine residue pairs that have an SASD below the maximum bound of the cross-linker (32 Å). The maximum bound of 32 Å was reached by scanning the experimental benchmark performance using *NoV* at different maximum bounds. The precision peaks at 32 Å (Figure S3). Models and cross-links were visualised using the molecular graphics program Chimera (Pettersen et al., 2004).

3D-EM Data

The function we used to score the models based on their fit to simulated 3D-EM density was taken from γ-TEMPy – a genetic algorithm for the simultaneous fitting of components in 3D-EM maps. We used the fitness function from that algorithm (F-score) which combines a mutual information (MI) score and a clash penalty score (PS):

$$F = (n \times MI) - PS$$

where n refers to the number of subunits. The MI score calculates how well a simulated map fits the experimental data (Vasishtan and Topf, 2011). The PS is a term to penalize for clashes. Further details can be found in the original paper (Pandurangan et al., 2015). The combination of cMNXL and F-score is as follows:

$$Combined = F + (0.5 \times cMNXL)$$

The weighting of cMNXL was chosen as a result of a systematic scan of weightings between 0 and 2.

QUANTIFICATION AND STATISTICAL ANALYSIS

Measuring Model Accuracy

Models are assessed via mean Cα-RMSD (meanRMSD) calculated using an in-house script and MODELLER-v9.18 (Šali and Blundell, 1993). Iteratively, the whole model is superposed onto the native structure, one subunit at a time, and the Cα-RMSD is calculated. The meanRMSD is the mean value of all of these Cα-RMSD values. Models are also evaluated using the fnat criterion, i.e. the proportion of native inter-subunit residue interactions maintained in the model (Lensink and Wodak, 2013). Residues are considered to be interacting if any of their atoms are within 5 Å of each other. The fnat score was calculated using an in-house script.

Score Assessment

We assessed the effectiveness of our scoring function using Precision and Area-Under-Curve (AUC) taken from the Receiver-Operating-Characteristic (ROC) curve. Precision is calculated as TP/(TP + FP), where TP (True Positive) is defined as a model that is scored in the top 10 by the scoring function and has a meanRMSD of $\leq$ 4 Å *and* an fnat score of $\geq$ 0.3, and FP (False Positive) is defined as a model that is scored in the top 10 by the scoring function but has a meanRMSD > 4 Å *or* fnat $\leq$ 0.3. The False Positive Rate (FPR) is calculated as FP/(FP+TN) where TN (True Negative) is defined as a model that is not scored in the top 10 by the scoring function and has a meanRMSD > 4 Å *or* fnat $\leq$ 0.3. The fnat criteria matches the criteria for a medium model in CAPRI (Lensink and Wodak, 2010). In the case where there are more than 10 models with the same top score, 10 models are randomly sampled from those models and the precision is calculated. This process is then repeated 1000 times to bootstrap an average precision.

ROC curves were calculated with a model being defined as Positive if the meanRMSD is $\leq$ 4 Å *and* fnat $\geq$ 0.3. ROC systematically lowers the scoring threshold to assess whether a model is a true positive, instead of the top 10 ranked models. ROC and AUC calculations were calculated using the ROCR package in R (https://www.r-project.org/).

Calculating Statistical Significance

The statistical significance between the precision and AUC of different scoring methods was calculated using a one-sided paired t-test, as implemented in R (www.r-project.org/). For example, the precision values of each benchmark case for the NoV score were compared directly to the precision values for each benchmark case for cMNXL (i.e. each case was *paired* together instead of simply combining them into one distribution). The alternative hypothesis was that the mean of the precision of cMNXL was greater than the mean of the precision of the NoV score (i.e. *one-sided*). In all cases, a significance threshold p-value of 0.05 was used, however p-values were often much smaller. Relevant p-values have been included in the main text.

DATA AND SOFTWARE AVAILABILITY

The experimental cross-links used to score the experimental benchmark can be found in the Supplemental Information. cMNXL is freely available to download from http://topf-group.ismb.lon.ac.uk/Software.html. The two benchmarks (theoretical and experimental) can be found in the Supplemental Information and in http://topf-group.ismb.lon.ac.uk.

Structure, Volume *26*

Supplemental Information

Modeling Protein Complexes Using Restraints from Crosslinking Mass Spectrometry

Joshua Matthew Allen Bullock, Neeladri Sen, Konstantinos Thalassinos, and Maya Topf

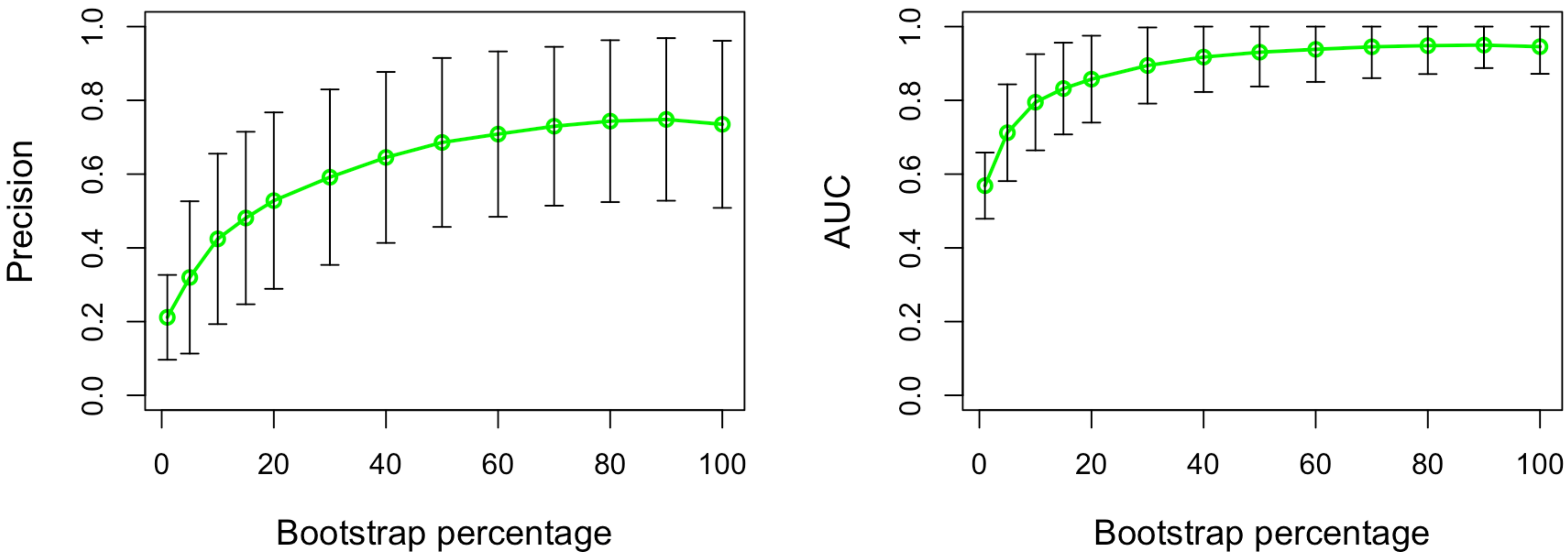

Figure S1 | *Relating to section Performance of cMNXL.* The effect of experimental crosslink recovery on performance. Bootstrapped performance of the benchmark in terms of (A) precision and (B) AUC change as crosslink recovery increases.

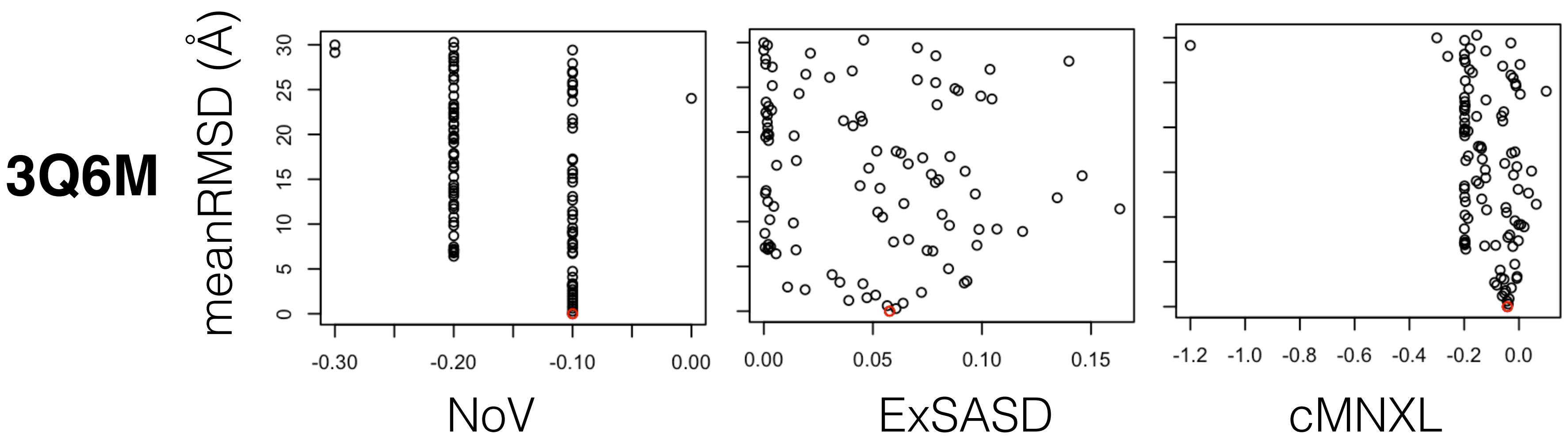

Figure S2 | *Related to section Performance of cMNXL.* Performance plots of 3Q6M showing the general poor performance of each scoring function. The precision of NoV_{inter} is skewed due to many models scoring the same, therefore giving it a high False Positive Rate

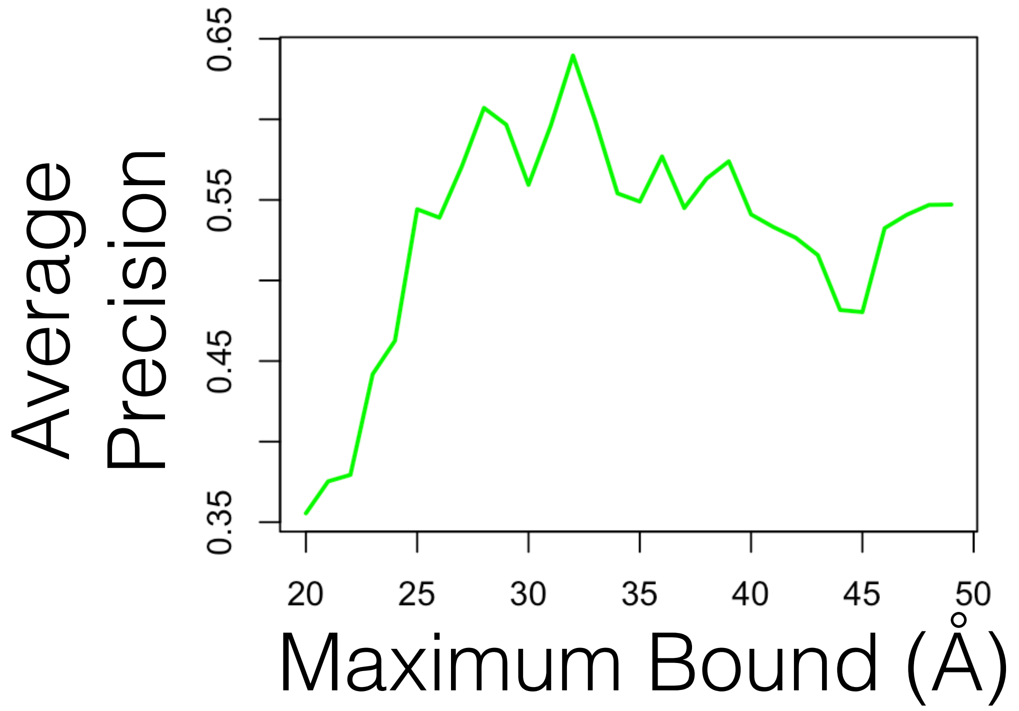

Figure S3 | *Related to section Experimental and Theoretical Cross-links.*
NoV score performance on the experimental benchmark at different maximum bounds. Peak precision is observed at 32Å

PDB	NoNA$_{intra}$			NoNA$_{inter}$			NoV			ExSASD			cMNXL			NoV$_{ED}$		ExED		cMNXL$_{ED}$	
	Prec	AUC	FPR	Prec	AUC	FPR	Prec	AUC	FPR	Prec	AUC	FPR	Prec	AUC	FPR	Prec	AUC	Prec	AUC	Prec	AUC
**3Q6M**	0.092	0.511	0.898	0.125	0.500	0.864	0.200	0.786	0.427	0.000	0.534	0.114	0.000	0.679	0.114	0.200	0.714	0.000	0.482	0.000	0.617
**3DFQ**	0.206	0.537	0.883	0.326	0.531	0.844	0.677	0.940	0.115	0.800	0.959	0.026	0.800	0.966	0.026	0.761	0.950	1.000	0.999	1.000	1.000
**1I10**	0.201	0.531	0.872	0.322	0.506	0.846	1.000	0.997	0.000	1.000	0.999	0.000	1.000	0.999	0.000	1.000	1.000	1.000	1.000	1.000	1.000
**1UJZ**	0.142	0.534	0.860	0.147	0.534	0.860	0.384	0.843	0.333	0.500	0.927	0.058	0.500	0.927	0.058	0.174	0.669	0.200	0.839	0.300	0.865
**2PSN**	0.100	0.524	0.103	0.231	0.596	0.816	0.635	0.955	0.102	0.800	0.969	0.023	0.500	0.940	0.057	0.477	0.927	0.400	0.915	0.300	0.884
**1IRI**	0.182	0.506	0.877	0.290	0.500	0.852	0.900	0.963	0.012	0.600	0.935	0.049	0.900	0.962	0.012	0.750	0.955	0.400	0.910	0.300	0.918
**1F05**	0.115	0.533	0.875	0.116	0.511	0.875	0.299	0.742	0.157	0.100	0.633	0.102	0.200	0.732	0.091	0.482	0.917	0.000	0.512	0.300	0.891
**1U8F**	0.310	0.525	0.846	0.316	0.531	0.846	1.000	1.000	0.000	1.000	0.999	0.000	1.000	1.000	0.000	1.000	1.000	1.000	0.998	1.000	1.000
**1JEQ**	0.390	0.500	0.900	0.475	0.514	0.817	0.809	0.936	0.098	0.900	0.982	0.000	0.900	0.970	0.000	0.716	0.868	1.000	0.941	1.000	0.950
**Avg.**	0.193	0.522	0.790	0.261	0.525	0.847	0.656	0.907	0.138	0.633	0.882	0.041	0.644	0.908	0.040	0.618	0.889	0.556	0.844	0.578	0.903

Table S2 | *Related to Figure 2*
Precision, AUC and FPR (False Positive Rate) for each benchmark case in the Experimental benchmark when scoring with cMNXL score and each constituent scoring term using both ED and SASD.

PDB	cMNXL		F Score (10 Å)		combined (10Å)		F Score (15Å)		combined (15Å)		F Score (20Å)		combined (20Å)	
	Prec	AUC	Prec	AUC	Prec	AUC	Prec	AUC	Prec	AUC	Prec	AUC	Prec	AUC
**3Q6M**	0.000	0.679	1.000	0.997	1.000	0.997	1.000	0.999	1.000	0.996	1.000	0.998	1.000	0.996
**3DFQ**	0.800	0.966	1.000	0.992	1.000	0.999	1.000	0.996	1.000	0.999	1.000	0.993	1.000	0.999
**1I10**	1.000	0.999	1.000	1.000	1.000	1.000	1.000	1.000	1.000	1.000	1.000	1.000	1.000	1.000
**1UJZ**	0.500	0.927	0.600	0.784	0.600	0.859	0.500	0.702	0.500	0.795	0.500	0.616	0.500	0.726
**2PSN**	0.500	0.940	0.900	0.850	0.900	0.936	0.900	0.859	0.800	0.938	0.800	0.856	0.800	0.939
**1IRI**	0.900	0.962	0.900	0.909	0.900	0.956	0.900	0.908	0.900	0.954	0.900	0.904	0.900	0.956
**1F05**	0.200	0.732	0.600	0.857	0.700	0.875	0.600	0.819	0.500	0.849	0.500	0.781	0.500	0.812
**1U8F**	1.000	1.000	1.000	1.000	1.000	1.000	1.000	1.000	1.000	1.000	1.000	1.000	1.000	1.000
**1JEQ**	1.000	0.975	1.000	0.944	1.000	0.976	1.000	0.943	1.000	0.973	1.000	0.940	1.000	0.972
**Avg.**	0.656	0.909	0.889	0.926	0.900	0.955	0.878	0.914	0.856	0.945	0.856	0.899	0.856	0.933

Table S4 | *Related to Figure 7*
Precision and AUC of cMNXL, F-score at 10Å, 15 Å and 20 Å resolution, and the combined score for each case in the Experimental benchmark.

PDB file	Modifications
3Q6M	Chain C - res 615 inserted to make identical to other chains
3DFQ	None
1I10	None
1UJZ	None
2PSN	None
1IRI	None
1F05	None
1U8F	None
1JEQ	437-454(A), 430-483(B), 269-323(B) removed

Table S5 | *Related to section Protein Complex Benchmarks.* Modifications made to each protein in the benchmark to facilitate model generation.
